# Biological receptor-inspired flexible artificial synapse based on ionic dynamics

**DOI:** 10.1038/s41378-020-00189-z

**Published:** 2020-09-07

**Authors:** Qifeng Lu, Fuqin Sun, Lin Liu, Lianhui Li, Yingyi Wang, Mingming Hao, Zihao Wang, Shuqi Wang, Ting Zhang

**Affiliations:** 1 i-Lab, Key Laboratory of Multifunctional Nanomaterials and Smart Systems, Suzhou Institute of Nano-Tech and Nano-Bionics (SINANO), Chinese Academy of Sciences (CAS), 398 Ruoshui Road, 215123 Suzhou, PR China; 2 Department of Health and Environmental Sciences, Xi’an Jiaotong Liverpool University, 111 Ren’ai Road, 215123 Suzhou, PR China; 3 Center for Excellence in Brain Science and Intelligence Technology, Chinese Academy of Sciences, 200031 Shanghai, PR China

**Keywords:** Nanoscale devices, Other nanotechnology

## Abstract

The memristor has been regarded as a promising candidate for constructing a neuromorphic computing platform that is capable of confronting the bottleneck of the traditional von Neumann architecture. Here, inspired by the working mechanism of the G-protein-linked receptor of biological cells, a novel double-layer memristive device with reduced graphene oxide (rGO) nanosheets covered by chitosan (an ionic conductive polymer) as the channel material is constructed. The protons in chitosan and the functional groups in rGO nanosheets imitate the functions of the ligands and receptors of biological cells, respectively. Smooth changes in the response current depending on the historical applied voltages are observed, offering a promising pathway toward biorealistic synaptic emulation. The memristive behavior is mainly a result of the interaction between protons provided by chitosan and the defects and functional groups in the rGO nanosheets. The channel current is due to the hopping of protons through functional groups and is limited by the traps in the rGO nanosheets. The transition from short-term to long-term potentiation is achieved, and learning-forgetting behaviors of the memristor mimicking those of the human brain are demonstrated. Overall, the bioinspired memristor-type artificial synaptic device shows great potential in neuromorphic networks.

## Memristors: Bio-inspired synapse

Taking inspiration from G-protein-linked receptors in biological cells, an artificial synapse is created for neuromorphic computing. Memristors are promising device units for the construction of neuromorphic computing platforms. Here, a team led by Ting Zhang from the Suzhou Institute of Nano-Tech and Nano-Bionics take inspiration from nature to fabricate a double-layer memristor. Specifically, they create a device that replicates the transmission of neural signals by ligands within G-protein-linked receptors. Their device includes a chitosan top-layer and a reduced graphene oxide bottom layer, with protons acting as charge carriers. Their device emulates some of the basic function of a synapse, including short-and long-term potentiation, spike-rate-dependent plasticity and learning/forgetting behavior.

## Introduction

Inspired by the massive parallelism, robust computation, fault tolerance, and energy efficiency of the human brain, neuromorphic computing has attracted a tremendous upsurge of research interest since synaptic behaviors were mimicked by Mead in 1996 using a floating-gate silicon metal-oxide-semiconductor transistor^1–5^. The implementation of neural networks at the software level with algorithms, such as Google’s Alpha Go and IBM Watson, based on complementary metal-oxide semiconductor technology requires a large amount of resources^6^. In addition, the tremendous amount of data processing between the memory and the central processing unit with a conventional von Neumann architecture limits the speed of current computer systems^7^. However, the human brain, containing ~10^11^ neurons and 10^15^ synapses, only consumes less than 20 W, making it more efficient than any other computing system^8^. Therefore, a computing system that can potentially emulate the neuromorphic network and overcome the von Neumann bottleneck is critically required. Memristors acting as a synapse unit, resembling their biological counterpart in terms of structure, can emulate several functions of the synaptic behaviors of the human brain. In addition, memristor-based artificial synapses can be used as basic building blocks to achieve large-scale neural network parallelism.

Thus, a great amount of attention has been attracted and much effort has been made to improve the performance of memristors. Generally, memristors are a type of two-terminal device with a layer of memristive material sandwiched between metal electrodes in either a vertical or planar structure. The working mechanism of the devices is highly related to the selection of both memristive and electrode materials. Recently, binary metal oxides, oxide perovskites, polymers, and 2D materials have been widely used as memristive layers in the construction of memristors^9–12^. Each category of material has advantages in terms of the working mechanism and the material properties, which is beneficial to applications in specific areas with the suitable selection of the materials. According to different working mechanisms, memristors can be categorized into filament-type and barrier-type devices^8^. With regard to filament-type memristors, the conductance of the devices is controlled by the formation and rupture of the filament. Undesired abrupt conductance changes and cycle-to-cycle variation are observed due to the random distribution of the filaments in the insulator caused by the stochastic nature of ion migration^13^. Different from filament-type memristors, barrier-type memristors generally modulate the conductance states by the defect effect, which can overcome the electroforming randomness, ensuring reproducibility, and device-to-device uniformity^14,15^. In addition, a series of continuous conductance states capable of emulating biosynaptic functions can be modulated by external stimuli. Therefore, use of the barrier-type memristor is considered a more suitable approach to emulate synaptic behaviors.

Based on the above analysis, the design of memristive devices, whose conductance states can be modulated by the defect effect, is a strategy to emulate biorealistic synaptic behaviors. In addition, the high efficiency of information processing and transmission in biological nervous systems provides an approach to designing memristive devices with high performance based on the metrics of biological systems. For example, a double-layer memristive device can be conceived based on inspiration from the working mechanism and structure of the G-protein-linked receptors of biological cells^16,17^, in which neural signals are transmitted by ligands from the extracellular environment in conjunction with receptors from transmembrane proteins located in the cytomembrane. In the designed device, mobile carriers provided by the top layer are able to mimic the functions of the ligands from the extracellular environment, while functional groups and defects in the bottom layer can behave like the receptors from transmembrane proteins. However, to the best of our knowledge, emulation of synaptic behaviors using this bioinspired working mechanism has not been reported even though the use of various materials, such as binary oxides, polymers, and perovskites, has been attempted in the fabrication of memristive devices^9,11,18,19^.

Herein, we designed and fabricated a double-layer memristive device in which chitosan (CS), an abundant, nontoxic, biodegradable, and biocompatible polymer, was selected as the top-layer material due to its electronic insulation and proton conduction properties^20^. The protons, hopping between the channel layers, behave like the ligands of biological cells. Reduced graphene oxide (rGO) nanosheets were used as the bottom-layer material, and the functional groups and defects in the nanosheets behave like the receptors of biological cells. The number of functional groups and defects in the rGO nanosheets can be well controlled by tuning the degree of the chemical redox reaction^21,22^. As a result, the device exhibits history-dependent memristive behaviors with continuous changes in the conductance states, meeting the fundamental requirements for mimicking the functions of a biological synapse. Consequently, a series of synaptic characteristics, including short-term potentiation (STP), long-term potentiation (LTP), spike-rate-dependent plasticity (SRDP), and learning-forgetting behaviors, toward biorealistic synaptic emulation were successfully realized.

## Results and discussion

### Device structure and properties

**Fig. 1:**
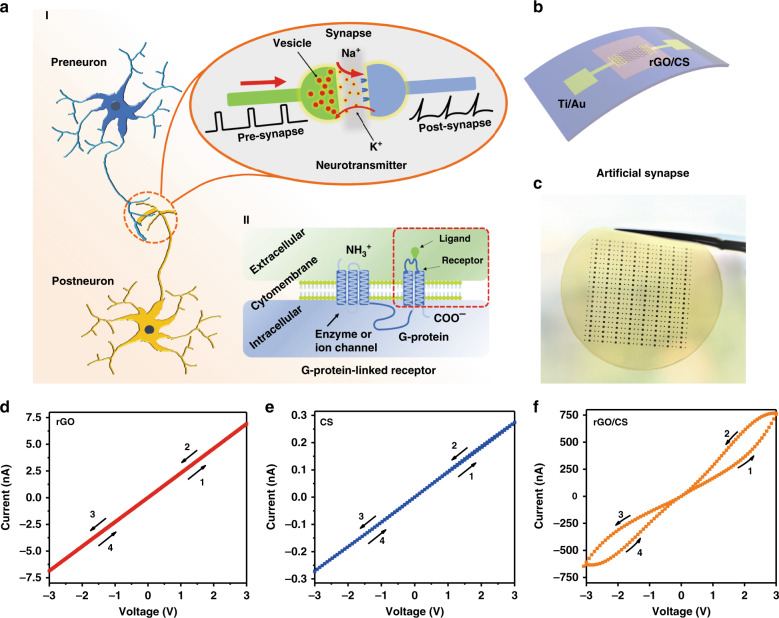
Structures and working mechanism of biological synapses and electrical properties of the receptor-inspired counterpart. **a**-I Schematic diagram for the connection of two neurons by a synapse in the biological system and **a**-II the working principle of the G-protein-linked receptor. **b** Schematic diagram and **c** optical image of the flexible memristors. CS and rGO were used as the top and bottom layers of the device channels, respectively. IV characteristics for the devices with **d** rGO, **e** CS, and **f** rGO covered by CS as the channel materials

Figure 1a shows the schematic diagrams for two neurons connected by a synapse in the nervous system and the corresponding working mechanism. Transmission of synaptic signals between neurons is a complex process that starts with the opening of voltage-controlled ion channels by external stimuli^23^. In brief, the diffusion of Ca^2+^ ions leads to the release of neurotransmitters from synaptic vesicles, and the ions bind to the receptor sites of Na^+^-gated ion channels at postsynaptic neurons. As a result, the Na^+^-gated ion channels will open, and Na^+^ can diffuse inside the cell, which leads to a positively charged membrane potential. The neuron will fire an action potential once the membrane potential reaches the threshold. This process contributes to the information flow and signal processing of the nervous system^24^. Based on the structure and types of receptors (acceptors), the synapses in the biological system can be classified into various categories. Taking the G-protein-linked receptor as an example, the ligands from the extracellular environment react with the receptors from transmembrane proteins located in the cytomembrane (circled by the red dashed lines in Fig. 1a-II), and an ionic channel is formed to transmit the signals. Inspired by the working principle of the G-protein-linked receptor, a double-layer memristive device consisting of rGO and CS as bottom-layer and top-layer materials, respectively, was fabricated, as shown in Fig. 1b. In the designed memristive device, the protons in CS and the functional groups in rGO are able to imitate the functions of the ligands and receptors of biological cells, respectively. Thus, the synaptic behavior can be mimicked using this device by monitoring the conductance change of the memristor. The detailed fabrication process and related parameters are included in Supplementary Figs. S1–S3. In addition, to emulate the neuromorphic functions of the brain, a matrix of the designed double-layer memristor was fabricated, and the optical image is presented in Fig. 1c. After fabrication of the devices, the current–voltage (IV) characteristics of the devices with rGO, CS, and rGO covered with CS as the channel materials were investigated, as presented in Fig. 1d, e, f, respectively. No hysteresis in the IV curves is observed for the devices with rGO or CS as the channel material, and the conductance is independent of the applied voltage. Regarding the device with rGO/CS as the channel material, a hysteretic IV characteristic is obtained, which is a main feature of memristive devices. The results imply the occurrence of an interaction between the CS membrane and rGO nanosheets, which leads to the change in device conductance being dependent on the applied voltages. In addition, the bend test shown in Supplementary Fig. S4 indicates the flexibility and mechanical reliability of the devices.

### Physical and electrical characteristics

**Fig. 2:**
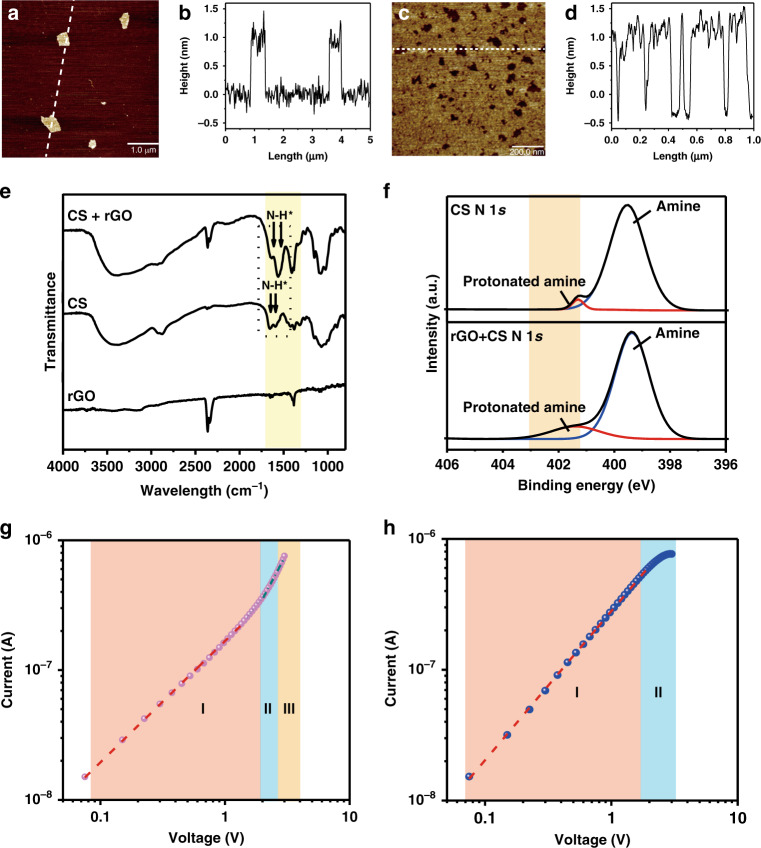
Characterization of the materials for the fabrication of the artificial synapses and the underlying mechanism for the memristive behaviors. **a**–**d** AFM images and the corresponding heights of rGO nanosheets. rGO nanosheets with an average size of ~1 μm were obtained. **e** Comparison of FTIR spectra for rGO, CS, and rGO/CS samples. Changes arising from antisymmetric –NH^3+^ deformation and –N–H bending vibrations were observed. The (**f**) N 1*s* peak in the XPS spectra shows that extent of protonation of amine groups increases from 2.96 to 14.25% for CS and rGO/CS samples. **g**, **h** demonstrate the forward and reverse IV curves of the memristor plotted in log scale, which are governed by SCLC

To explore the underlying mechanism of the memristive behaviors of the device, the physical and electrical properties of the rGO nanosheets, the CS membrane and the interactions between them were investigated in detail. From the atomic force microscopy (AFM) images in Fig. 2a–d, there are many nanoholes in the rGO nanosheets, with an average size of ~1 μm, which lead to a number of defects (more information about the size and thickness of the nanosheets is presented in Supplementary Figs. S5 and S6). In this research, since the channel length of the fabricated device is 40 μm and the size of the rGO nanosheets is ~1 μm, it can be inferred that there are a number of homojunctions caused by overlaps of the nanosheets and intersecting grain boundaries in the channel. The homojunctions and grain boundaries as well as the defects inside the rGO nanosheets work as traps and impede the flow of current through the channel. By contrast, from the XPS spectra shown in Supplementary Fig. S7, a considerable number of functional groups are observed in the rGO nanosheets, which will contribute to the current induced by the hopping of the carriers in the channel^25–27^. In addition, from the comparison of the FTIR spectra for CS and rGO/CS in Fig. 2e, obvious changes in the bands at 1628–1633 cm^−1^ and 1524–1529 cm^−1^ are observed; these bands are assigned to amide I and antisymmetric –NH^3+^ deformation and to amide II and –N–H bending vibrations, respectively^28^. Thus, the changes are considered to be caused by the protonation of amide, which is also supported by the N 1*s* peaks of the XPS spectra shown in Fig. 2f, indicating that the extent of protonation of amine groups increases from 2.96 to 14.25%. No obvious change in the C 1*s* peaks is observed for the XPS spectra collected from the CS and CS/rGO samples, as shown in Supplementary Fig. S8. Furthermore, the increased conductance of the devices with humidity (Supplementary Fig. S9) is additional evidence for the above analysis, as the extent of protonated amino groups of CS is influenced by the ambient humidity^29,30^.

Based on the above results, it can be inferred that the current in the channel is caused by the hopping of protons through the functional groups^27,31^. The hopping process resembles the interaction between the ligands and receptors in biological systems. In other words, the formation of current based on ionic dynamics in the memristor is similar to the working mechanism of G-protein-linked receptors. In contrast, traps formed by the grain boundaries, homojunctions, and defects will capture the carriers and impede the current, as mentioned above^32^. From the slopes of the IV curve in regions I and II of Fig. 2g, as the voltage increases, the current changes from the low-exponent space-charge-limited current (SCLC) affected by shallow traps to the high-exponent SCLC affected by deep traps. More injected carriers will fill the trapping sites in order of the deeper energy levels, as indicated in region III, with the further increase in voltage, which leads to the dramatic increase in conductance^33,34^. Because holes are the main carriers in this device, their injection, transport, and trapping processes are analyzed to provide a possible explanation for the underlying mechanism. The CS membrane serves as a proton reservoir, and the current is formed due to the hopping of protons through the functional groups in the channel. However, the traps in the rGO nanosheets acting as barriers will impede the current, and therefore, the resistance is relatively large in region I of the forward IV characteristic, as shown in Fig. 2g. As the voltage increases, protons captured at the trapping sites diminish the existing trap concentration gradually in the order of the energy levels, and the resistance decreases, which is supported by the increasing slopes of the fitting curves in regions II and III of Fig. 2g^34,35^. Thus, the modulation of conductance can be achieved as a function of the trapped carrier concentration. As a result, the conductance increases continuously depending on the history of operation, indicating the typical memristive property. In the reverse trace, protons in the shallow traps are released as the voltage decreases. However, protons trapped in the deep trapping energy sites cannot escape easily. Thus, a more conductive channel is obtained than that forward sweeping due to the unreleased carriers in the trapping sites. As a sequence, an obvious hysteretic loop is generated, as illustrated in Fig. 2f. In the reverse IV curves, another linear relation is fitted on the log scale, as shown in region I of Fig. 2h, which correlates with the lower-exponent SCLC. Therefore, it can be concluded that the physical mechanisms for memristive behaviors are mainly attributed to the SCLC transition caused by the charge trapping/detrapping process with different filling ratios of protons in the trapping sites of the rGO nanosheets as a function of the operation history.

### Working mechanism of the artificial synapse

**Fig. 3:**
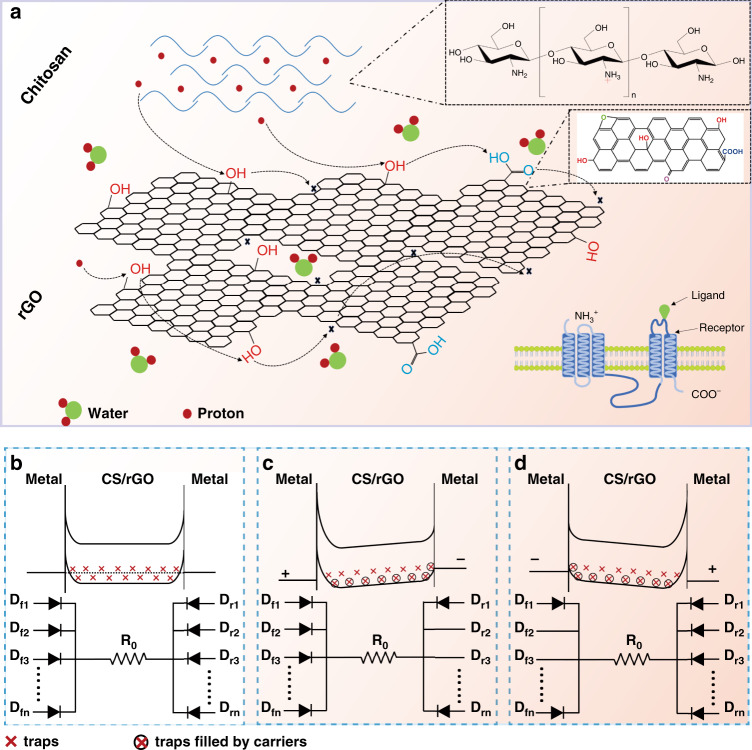
Proposed model for working mechanism of the artificial synapse. **a** Schematic diagram for the working mechanism of the memristor. The current is formed due to the hopping of protons provided CS through functional groups and limited by the traps in rGO nanosheets. **b**, **c**, **d** show a proposed model consisting of a number of face-to-face diodes for the memristor working in zero bias, forward bias, and reverse bias conditions, respectively

Based on the above discussion, a schematic diagram for the working mechanism of the memristor is demonstrated in Fig. 3a, and a corresponding model is proposed. The hopping process of protons through functional groups is similar to the interaction of ligands and receptors in biological cells, as indicated in the right corner of Fig. 3a. Protons from the CS membrane act as ligands from the extracellular environment, while the functional groups in the rGO nanosheets behave like the receptors from transmembrane proteins. The current induced by the hopping of protons in the device channel mimics the transmission of neuron signals. The band gap of the rGO nanosheets is estimated to be 3.5 eV from the UV-vis absorption spectra (Supplementary Fig. S10). Initially, it is tentatively hypothesized that the traps impede the carriers in the channel due to the barrier heights^32,36,37^. Thus, the memristor is modeled as a number of face-to-face diodes with a resistor between them, as shown in Fig. 3b. When a positive voltage is applied to the left terminal of the device, as presented in Fig. 3c, hopping of the protons through the functional groups occurs under the external electric field. A portion of the injected protons is captured by the trapping sites. With increasing applied voltage, the traps are gradually filled in the order of the energy levels. Then, the barrier heights (or the number of diodes in the proposed model) gradually decrease, as indicated in Fig. 3c, which leads to a decrease in the impedance. Therefore, the slope of the channel current is enhanced due to the decreased impedance. As a result, the forward trace labeled 1 in Fig. 3f is obtained. In the reverse sweep, the carriers trapped in the deep energy levels cannot be thoroughly released with decreasing applied voltage, which indicates that a portion of the traps are always filled and that the diminished barrier height cannot be recovered to the initial states. Thus, the reverse trace labeled 2 in Fig. 3f with a current larger than the forward trace is achieved. The same working principle is also applicable to the case of a negative sweep of the IV curves, as shown in Fig. 3d.

### Synaptic behaviors of the artificial synapse

**Fig. 4:**
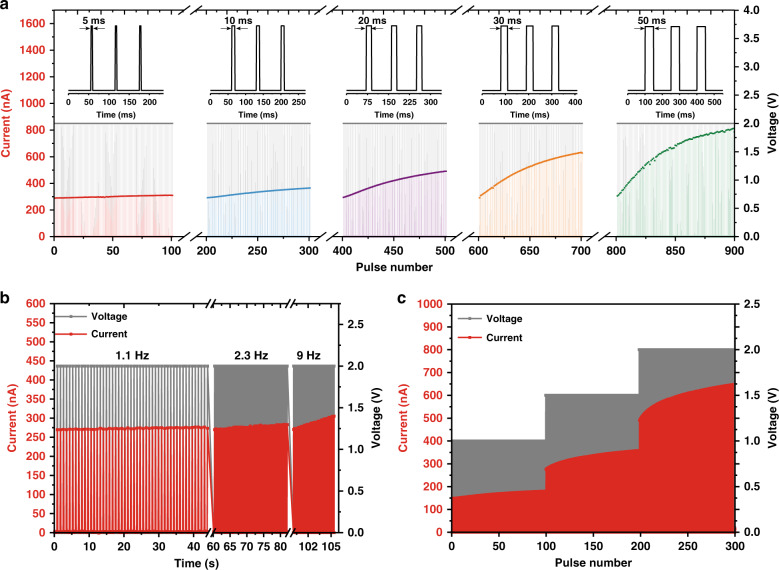
Synaptic behaviors of the artificial synapse. **a** Plasticity characteristics of the artificial synapse by 100 consecutive pluses with pulse widths of 5, 10, 20, 30, and 50 ms. **b**, **c** demonstrate the SRDP plasticity behavior of the artificial synapse

After understanding the underlying mechanism of the memristor, a possible electronic artificial synapse that can imitate the behaviors of biological synapses is demonstrated. Compared with a biological synapse, the two electrodes of the fabricated device are analogous to the presynaptic and postsynaptic terminals. The synaptic strength, or synaptic plasticity, in biological systems, which is believed to be a result of the number of neurotransmitters released at the synapse, is determined by presynaptic Ca^2+^ flux induced by an action potential^38,39^. Thus, a temporal change in synaptic strength can be achieved as a function of the stimuli. Similarly, the synaptic weight of an artificial synapse, which is defined by the conductance of the memristor, can be modulated by the successive stimuli of external applied pulses. Therefore, first, the plasticity characteristics of the artificial synapse by 100 consecutive pulses with an amplitude of 2 V and pulse widths of 5, 10, 20, 30, and 50 ms were investigated, as presented in Fig. 4a. In the case of 100 successive stimuli with a pulse width smaller than 5 ms, no obvious increase in the response current, that is, synaptic potentiation, is observed. With increasing pulse width, an enhancement in synaptic potentiation is obtained. However, when the pulse width is larger than 50 ms, some fluctuations in the response current occur. This phenomenon is probably attributed to different facilitation ratios caused by the variations in relaxation time for each stimulus, which is dependent on the conductance mechanism, when the device is stimulated by pulse trains with a pulse width larger than 50 ms^40^. Therefore, a pulse width of 30 ms is selected to investigate other synaptic behaviors. The SRDP was examined by varying the frequency and amplitude of the pulses. For consecutive pulses with an amplitude of 2 V and a width of 30 ms, there is almost no increase in the response current at a given frequency of 1.1 Hz. However, when the frequency increases to 9 Hz, the current increase rate is larger than that of previous conditions with lower frequencies, as illustrated in Fig. 4b. This behavior is related to the competition between the relaxation time of the artificial synapse and the interval between two pulses. When the amplitude of pulses increases from 1 to 2 V, the current increase rate becomes even larger, as illustrated in Fig. 4c, which corresponds to the filling of traps with higher energy levels, as discussed above.

### Emulation of learning and forgetting process

If the external stimuli are removed after the potentiation of artificial synapses, the conductance will decay to its initial low value, dependent on the number of applied pulses. If the conductance decays to its initial value rapidly (within several seconds), it is termed STP^8^. The process is related to the release of trapped carriers in the trapping sites, and the relaxation follows the exponential law with time described by the equation below^31,41^:

1 $${I} = \left( {I_0 - I_\infty } \right)\exp \left[ { - \left( {\frac{t}{\tau }} \right)^\beta } \right] + I_\infty$$

where *t* is the relaxation time, *τ* is the time constant, *I*_0_ is the initial current level, *I*_∞_ is the current level at infinite time, and *β* is the stretch index, with a value between 0 and 1.

**Fig. 5:**
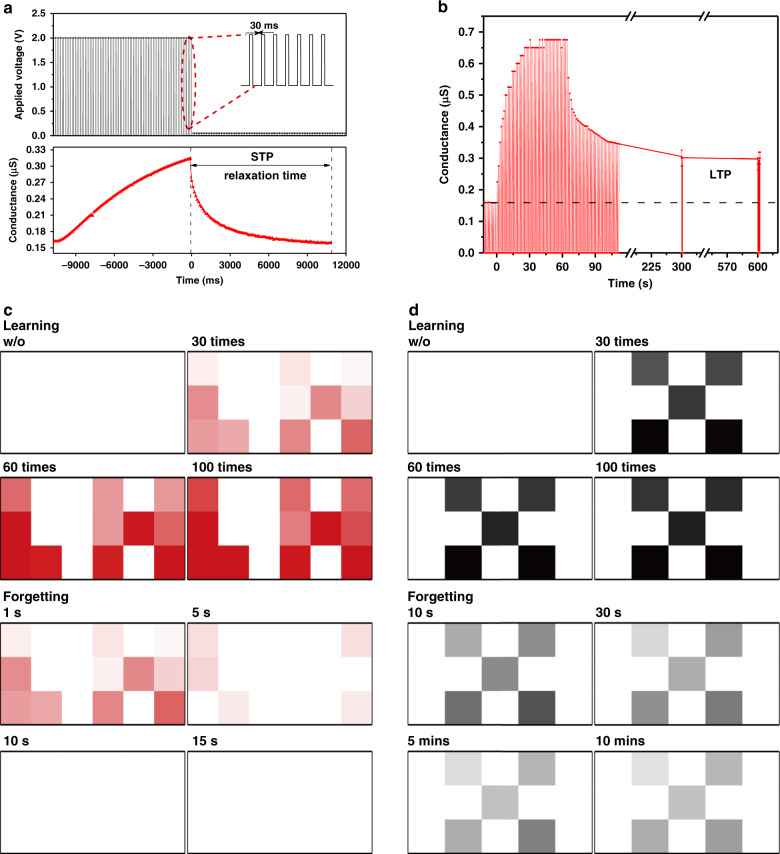
Emulation of learning and forgetting process with the designed artificial synapse. **a**, **b** show the STP and LTP behavior of the artificial synapse. A demonstration of STM and LTM of the artificial synapses by putting letters “LH” and “X” into the matrix is presented in (**c**) and (**d**)

In this research, an STP behavior with a relaxation time constant of 10.5 s is achieved after 100 consecutive pulses with an amplitude of 2 V and a pulse width of 30 ms, as shown in Fig. 5a. The STP is replicated in Supplementary Fig. S11 of the Supplementary materials, indicating the stability of the artificial synapse. In addition, STP can be trained into a permanent change in the conductance state called LTP. In electronic devices, the transition from STP to LTP is monitored by the decrease in conductance during the decay time. As shown in Fig. 5b, the conductance is maintained at a higher value than the initial state for a long time (more than 10 min) after the removal of the applied pulses, when the pulse width of stimuli increases to 500 ms. This phenomenon represents the transition from STP to LTP.

In biological systems, as the Ebbinghaus forgetting curve describes in the early period, information is lost quickly when it is initially learned, and the rate at which these memories are lost is dependent on the frequency the information is reviewed^42^. In addition, the multistore model of human memory proposed by Richard C. Atkinson and Richard Shiffrin in 1958 states that new information coming from outside the environment will be selected as short-term memory (STM) and long-term memory (LTM). Both studies indicate that the retention of the memory level is dependent on the training times and similar to the transition from STP to LTP in memristive devices demonstrated in this research^43^. Therefore, the artificial synapse developed in this study, capable of mimicking both STP and LTP and its transition process, has the potential for the realization of STM and LTM and, in turn, the construction of a neuromorphic network. To prove the ability of STM and LTM emulation, a simulation was implemented by inputting the letters “LH” and “X” into the memristor matrix, as shown in Fig. 5c. “LH” was stimulated by 100 consecutive pulses with an amplitude of 2 V and a pulse width of 30 ms, and “X” was trained with the same number of pulses but for a longer pulse width of 500 ms. Both “LX” and “X” were gradually memorized by the devices with increasing spike number, which represented the dynamic learning process. After learning 100 times, the applied stimuli were removed, and the forgetting process was investigated. For the devices stimulated by the pulses with a shorter pulse width, that is, 30 ms, the forgetting process was faster than that with a pulse width of 500 ms, and therefore, the “LX” was almost completely forgotten in 10 s, which was consistent with the relaxation time shown in Fig. 5a. Regarding the devices trained by pulses with a longer pulse width of 500 ms, the letter “X” can be memorized for more than 10 min, which can be considered LTM. The above demonstration indicates that basic learning and forgetting behaviors, which are performed by a complex neural network in the human brain, can be successfully emulated using the memristor matrix^44^. In addition, the continuous conductance states of the device modulated by the pulse stimuli provide a solid foundation for implementing the neural network at the hardware level. All the results imply that memristor-based artificial synapses can be regarded as building blocks of artificial neural networks, representing an essential step toward neuromorphic applications.

## Conclusions

In conclusion, a bioinspired double-layered memristor matrix was fabricated with rGO nanosheets prepared by chemical redox reactions and natural CS biopolymers. Distinct from previously reported memristors, the memristive device developed in this research is inspired by the working principle of the G-protein-linked receptors of biological cells, and the underlying mechanism is based on the interaction between rGO and CS. Studies on the physical and electrical properties of the devices and materials prove that the memristive behavior is due to the interaction between protons provided by CS and the defects and functional groups in the rGO nanosheets. Learning-forgetting processes based on the STP and LTP properties of the devices were demonstrated, which showed great potential in emulating the functions of the human brain. Overall, artificial synapses offer a new avenue for the construction of neuromorphic computation systems based on devices with bionic structures and working mechanisms.

## Materials and methods

### Synthesis of rGO aqueous solutions

Graphene oxide (GO) was exfoliated from natural graphite (350 mesh, Aladdin, China) by a modified Hummers method^45^. Then, a 10 μg/mL GO dispersion in aqueous solution was prepared by ultrasonication for 1 h. The obtained GO aqueous solution was mixed with 74 μL ammonia solution (28 wt% in water, Sinopharm, China) and 4 μL hydrazine solution (35 wt% in water, Wuxi Zhanwang, China) for chemical reduction of GO. The weight ratio of hydrazine to GO was ~6:10. After fully mixing, the flask was immersed into an oil bath at 90 °C for 1 h with magnetic stirring and cyclic cooling water. After complete reaction in the flask, an rGO dispersion was obtained, which was used as the precursor for channel materials of memristors without any further treatment.

### Fabrication of the memristors on flexible substrates

A diluted polyimide (PI) solution was spin-coated on cleaned glass and cured at 300 °C for 1 h. Then, electrodes (Ti/Au) were deposited by sputtering and patterned using a photolithograph and lift-off method on the PI substrates. Next, an aqueous rGO dispersion was drop-cast on the sample, and the active regions were defined using photolithography and O_2_ plasma etching. To reduce the contact resistance between the electrodes and rGO nanosheets, the sample was annealed at 150 °C for 90 min in ambient argon. Both IV characteristics of the device before and after annealing and the isolation between devices are illustrated in Supplementary Fig. S2. Afterward, the 2% CS solution (5 μL) was dropped on top of the active regions. Finally, the PI film was peeled off from the glass. The detailed fabrication process is demonstrated in Supplementary Fig. S1 of the Supplemental materials.

### Device characterizations

The size and surface morphology of rGO nanosheets were investigated by AFM (Bruker Dimension, USA) and scanning electron microscopy (SEM, Hitachi Regulus, Japan) at an accelerating voltage of 5 kV. The thickness of the rGO nanosheets could also be determined from the AFM results. FTIR spectra were collected via a Thermo Nicolet iN 10 spectrometer (Thermo-Fisher, USA) with the laser operating at 77 K in a liquid nitrogen environment. XPS analysis was carried out using a Thermo Scientific EXCALAB 250 XI system (Thermo-Fisher, USA). Raman spectra of the materials were collected using a Horiba-JY Labram HR system (HORIBA JY, France). A Perkin Elmer Lambda 25 spectrometer (Perkin Elmer, USA) was used to investigate the absorbance of rGO nanosheets. All the electrical measurements were performed at 25 °C and 40% humidity using an Agilent B1500A semiconductor parameter analyzer (Keysight, USA).

## Supplementary information

Supplementary Information
