# Supplementary material for: Biological receptor-inspired flexible artificial synapse based on ionic dynamics
Source: Microsyst Nanoeng. 2020 Sep 7;6:84. doi: 10.1038/s41378-020-00189-z (PMC8433456; doi:10.1038/s41378-020-00189-z)
Supplement: Supplementary file 1 — Supplementary Information [file 41378_2020_189_MOESM1_ESM.docx]

Biological Receptor Inspired Flexible Artificial Synapse Based on Ionic Dynamics

Qifeng Lu^1^, Fuqin Sun^1^, Lin Liu^2^, Lianhui Li^1^, Yingyi Wang^2^, Mingming Hao^1^, Zihao Wang^1^, Shuqi Wang^1^, and Ting Zhang^1*^

^1^ *i*-lab, Key Laboratory of multifunctional nanomaterials and smart systems, Suzhou Institute of Nano-Tech and Nano-Bionics (SINANO), Chinese Academy of Sciences (CAS). 398 Ruoshui Road, Suzhou, 215123, P. R. China.

^2^ Department of Health and Environmental Sciences, Xi’an Jiaotong Liverpool University, 111 Ren’ai Road, Suzhou, 215123, P. R. China.

These authors contributed equally: Qifeng Lu and Fuqin Sun.

[*] Corresponding author. E-mail: [tzhang2009@sinano.ac.cn](mailto:tzhang2009@sinano.ac.cn)

Fig. S1 shows the schematic diagrams for the fabrication of flexible memristors. Firstly, glass substrates were cleaned ultrasonically in acetone and [isopropanol](javascript:;) (IPA) for 15 minutes followed by the DI water rinsing for several cycles. The cleaned glass was dried by nitrogen and baked at 100 ℃ for 15 minutes before spin-coating of PI solution. The PI solution used in this research was diluted with NMP at a volume ratio being 5:2 to obtain a proper [viscosity](javascript:;). The diluted PI solution was spin-coated on the cleaned glass with spin speeds of 600 rmp for 6 s followed by 1100 rmp for 5 s. The coated sample was prebaked at 100 °C for 30 minutes and then, cured at 300 °C for one hour with a ramp rate of 10 °C per minute. Flexible PI substrates with a desired thickness (~ 14 μm) was obtained and can be peeled-off effortlessly from the glass after fabrication of the devices. Afterwards, Ti/Au electrodes with thicknesses of 10 nm and 50 nm, respectively, were deposited by sputtering and patterned using photolithograph and lift-off methods. Then, a UV treatment for 10 minutes was performed before drop-cast of rGO solutions at 50 °C. After solvent was fully evaporated, the sample was annealed at 120 °C for 90 minutes in argon ambient to reduce the contact resistance. After that, the photoresist was used to protect the channel region and the unprotected area was etched by oxygen plasma for 10 minutes with a power of 200 W and an O_2_ flow rate of 200 sccm at 80 mTorr. Finally, after removal of the photoresist, 2 wt% chitosan solution dissolved in 2 wt% acetic acid was covered on the top of the rGO nanosheets at 50 °C and baked for 30 minutes.


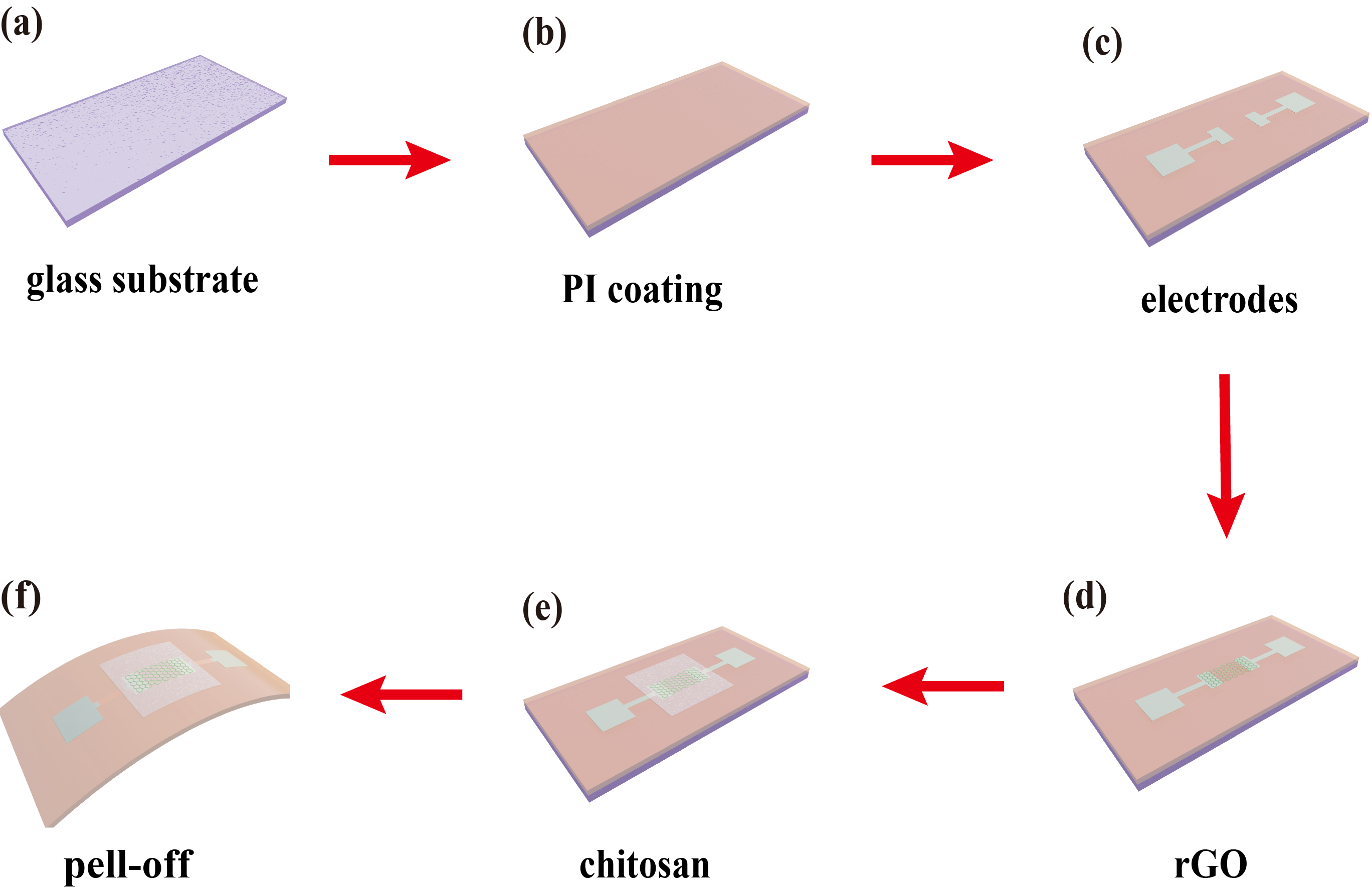


**Fig. S1** Fabrication process of the memristors investigated in this research.

Fig. S2a shows IV relationships of rGO channel after drop-casted for one, two and three times, labelled as rGO1, rGO2, and rGO3, respectively. The samples annealed at 150 ℃ for 90 minutes in argon ambient are labeled as rGO1_A, rGO2_A, and rGO3_A, respectively. It can be seen that negligible current is observed for rGO1 and rGO2. Although a current at the order of 0.1 nA is obtained for rGO3, there is a nonlinear relationship in the IV curves for the sample without annealing. This behavior is caused by the poor contact between rGO nanosheets and electrodes. After annealing, an Ohmic contact is obtained and the current increases dramatically. The increased current is mainly attributed to the improved contact rather than the possible reduction of rGO nanosheets during the annealing process, which is confirmed by the Raman spectra with the almost same D/G ratio of the rGO nanosheets before and after annealing as shown in Fig. S3. In addition, Fig. S2b shows that the devices are isolated from each other as indicated by the IV curve for rGO_A_etch, which implies that mesas are formed using oxygen plasma etching.


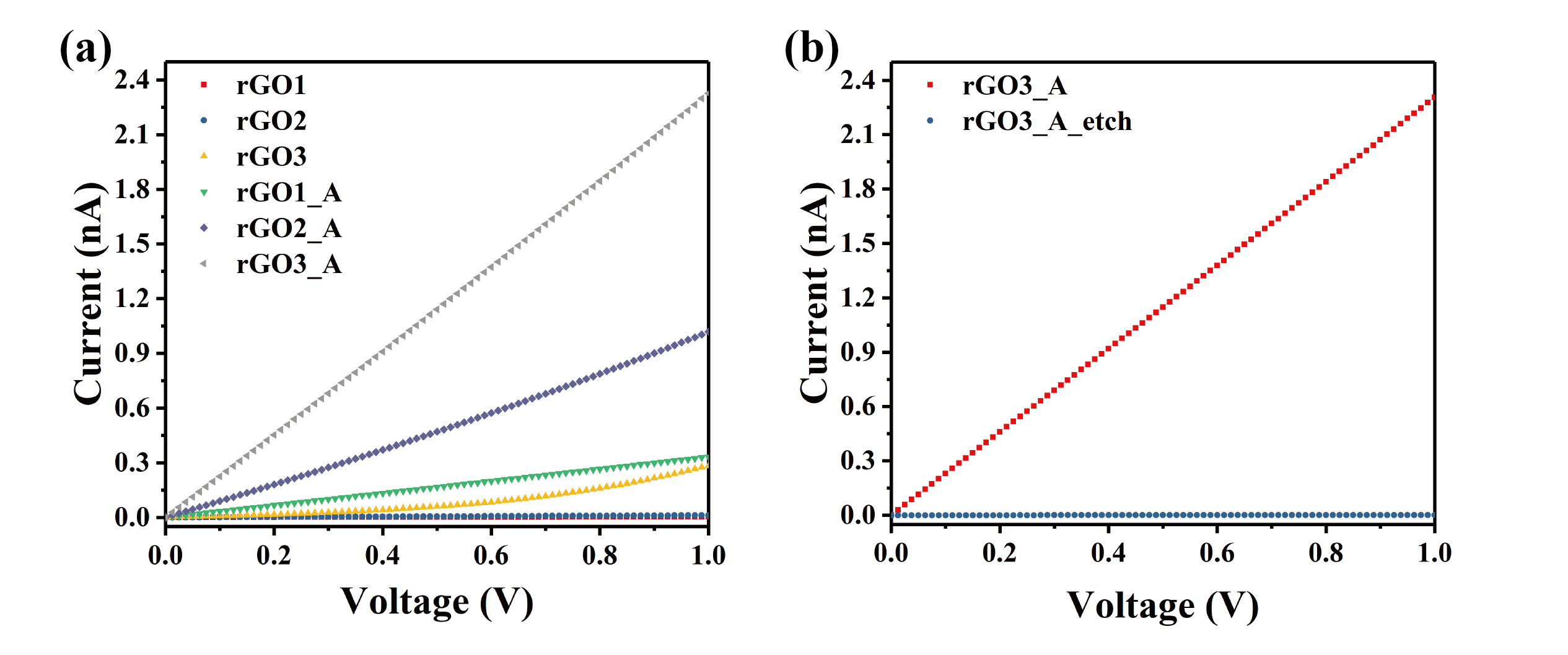


**Fig. S2 a** IV curves of the devices with rGO channel drop-casted for differents before and after annealing. **b** Almost no leakage current is observed between the devices after plasma etching.


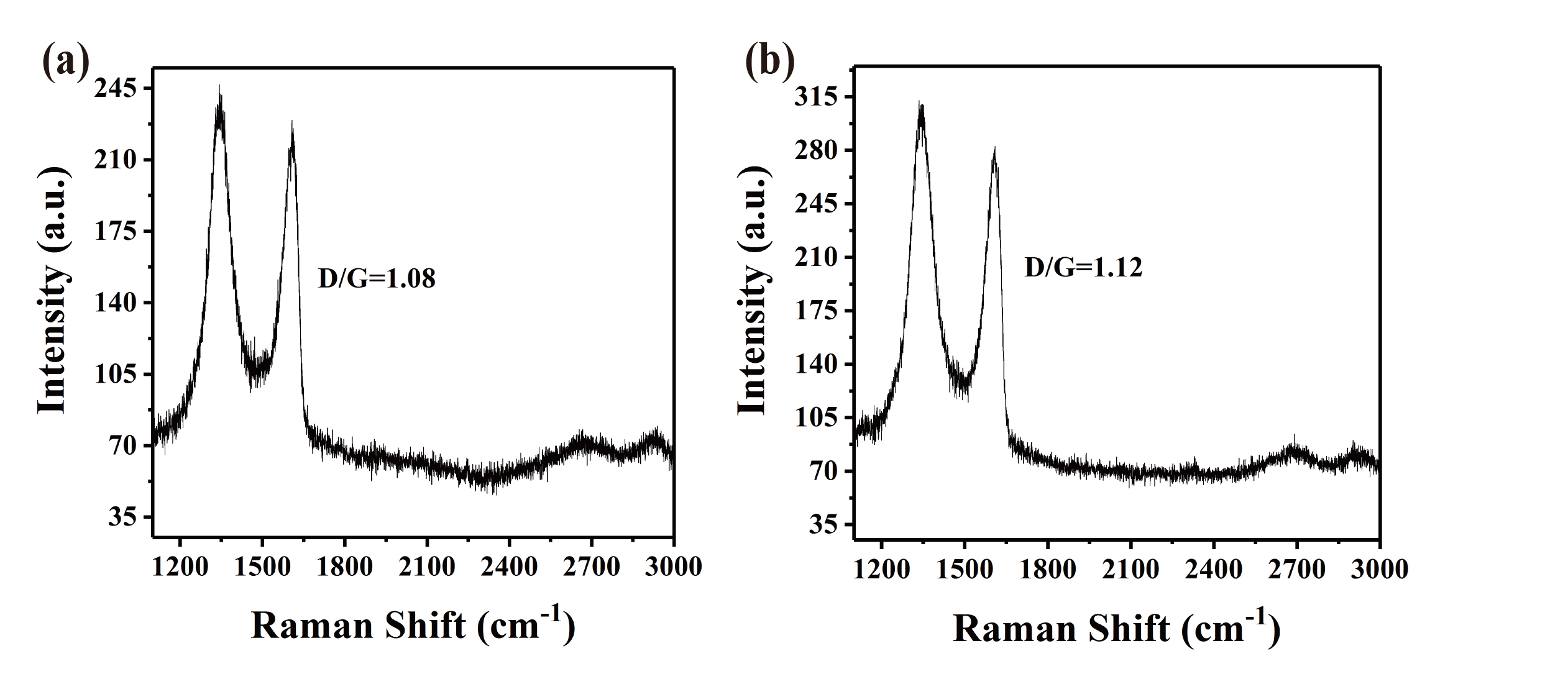


**Fig. S3** Raman spectra of rGO **a** before and **b** after annealing in argon ambient.

Fig. S4 compares IV characteristics of the artificial synapses in 1000 cycles of bending deformation. It is found that the devices on PI substrate were mechanically robust with almost no performance degradation due to bending effect.


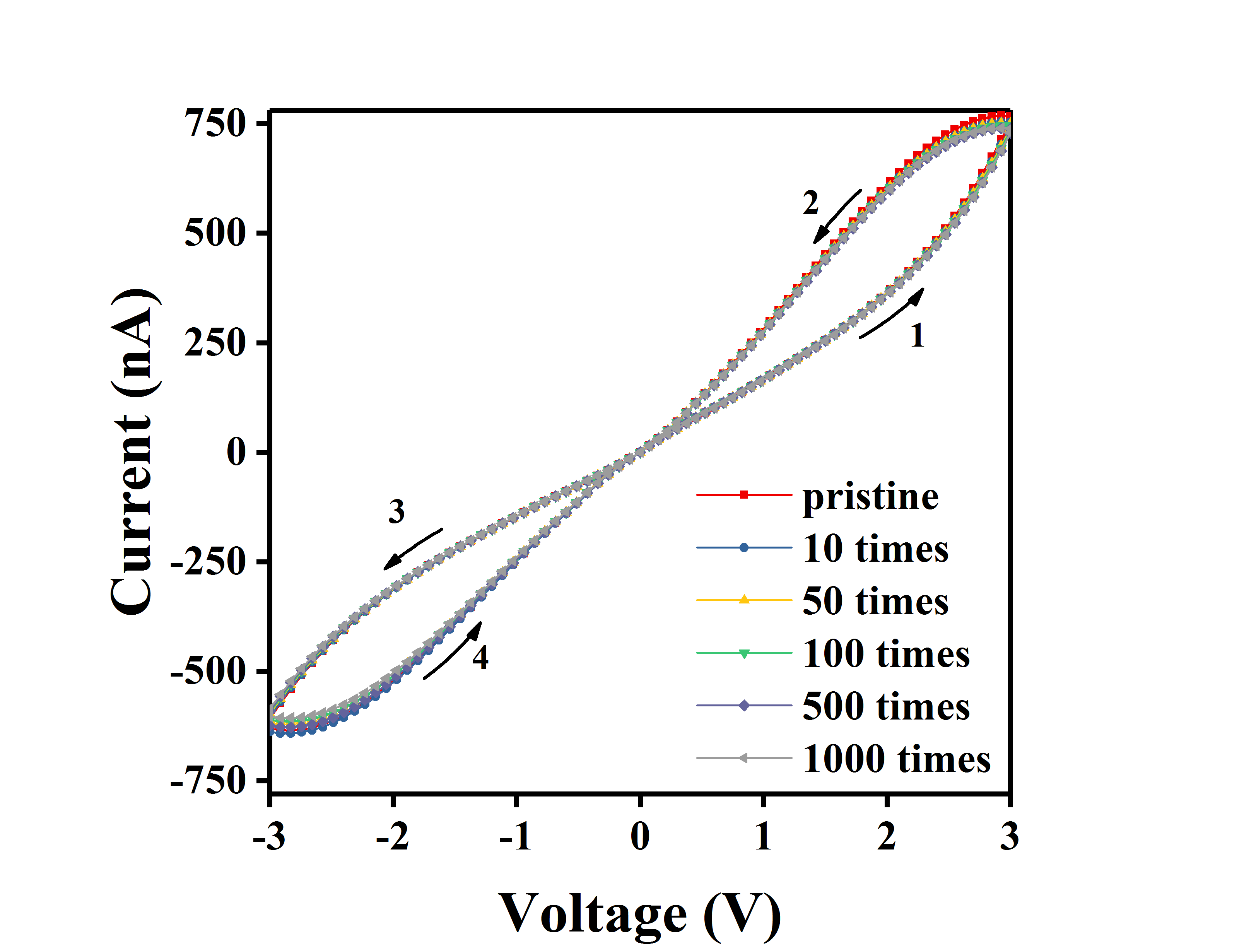


**Fig. S4** No obvious change in the IV characteristics was observed for the flexible artificial synapse after 1000 bending cycles.

From SEM images of rGO presented in Fig. S5, single or few layers rGO nanosheets with sizes ranging from 500 nm to 1 μm were obtained using the method described in this research. In addition, almost no impurity was introduced in the samples before and after annealing, since the aqueous was used as the solvent for the dispersion of nanosheets.


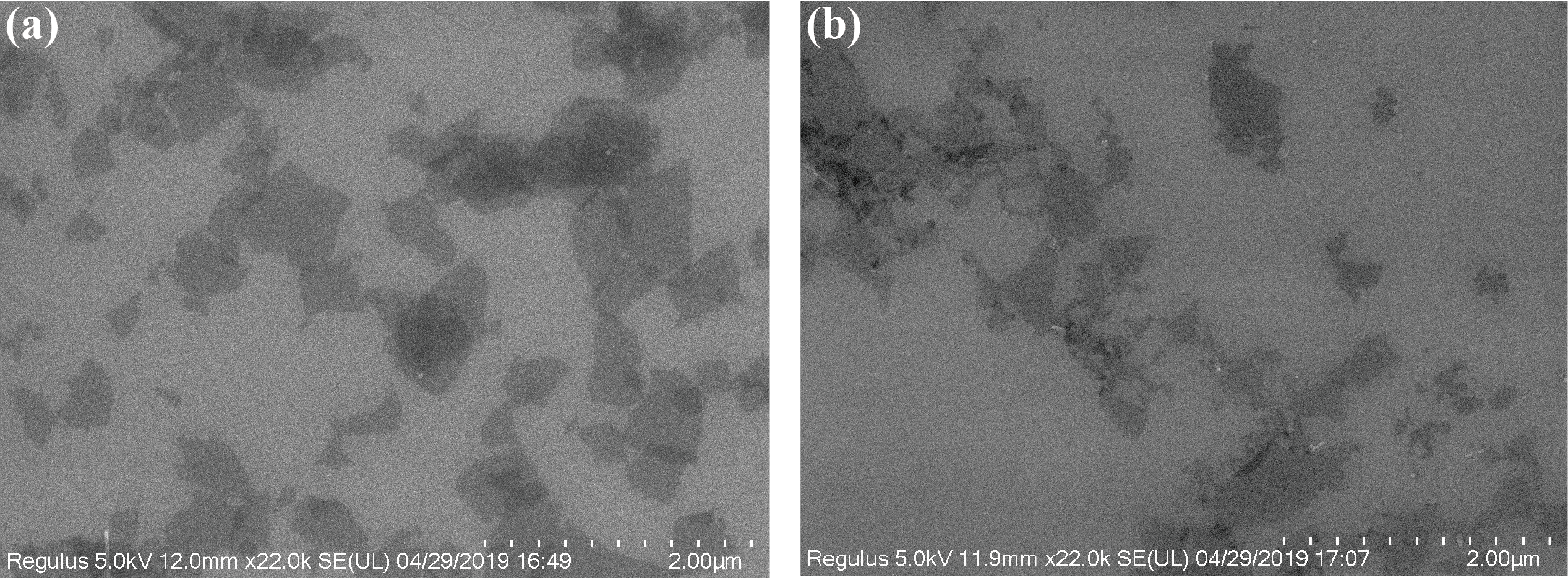


**Fig. S5** SEM images of rGO **a** before and **b** after annealing in argon ambient.

The AFM results shown in Fig. S5 confirms the thickness of the nanosheets being about 1 nm, which is consistent with the typical thickness of a single layer partially reduced graphene oxide .


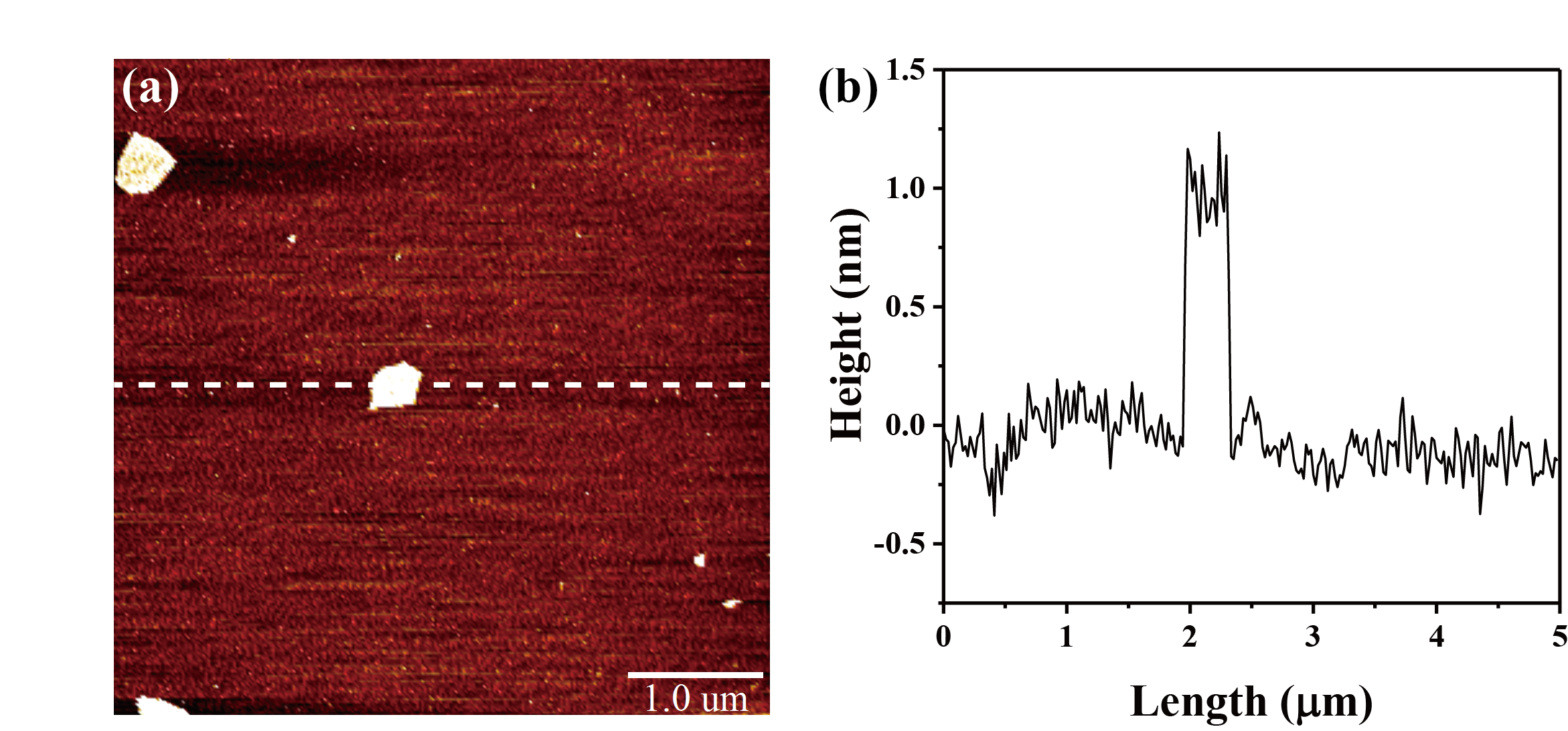


**Fig. S6** **a** An AFM image of rGO nanosheets and **b** the correspond height of the rGO.

Fig. S7 shows the XPS spectra for the C 1*s* peaks of rGO nanosheets investigated in this research. Two distinct C 1*s* peaks at the binding energies of 284.6 and 286.7 eV and two small C 1*s* peaks at 287.8 and 289.0 eV are observed. The peak at 284.6 eV is assigned to the nonoxygenated ring (C-C and C=C bonds), whereas the peak at 286.7 eV arises from the C in C-O bonds of the hydroxyl, ether, and epoxide functional groups. The two small peaks at 287.8 and 289.0 eV are attributed to the C=O bonds of the carbonyl (C=O) and carboxyl (COOH) functional groups, respectively. These functional groups will contribute to the hopping of the carriers in the channel as explained in the manuscript.


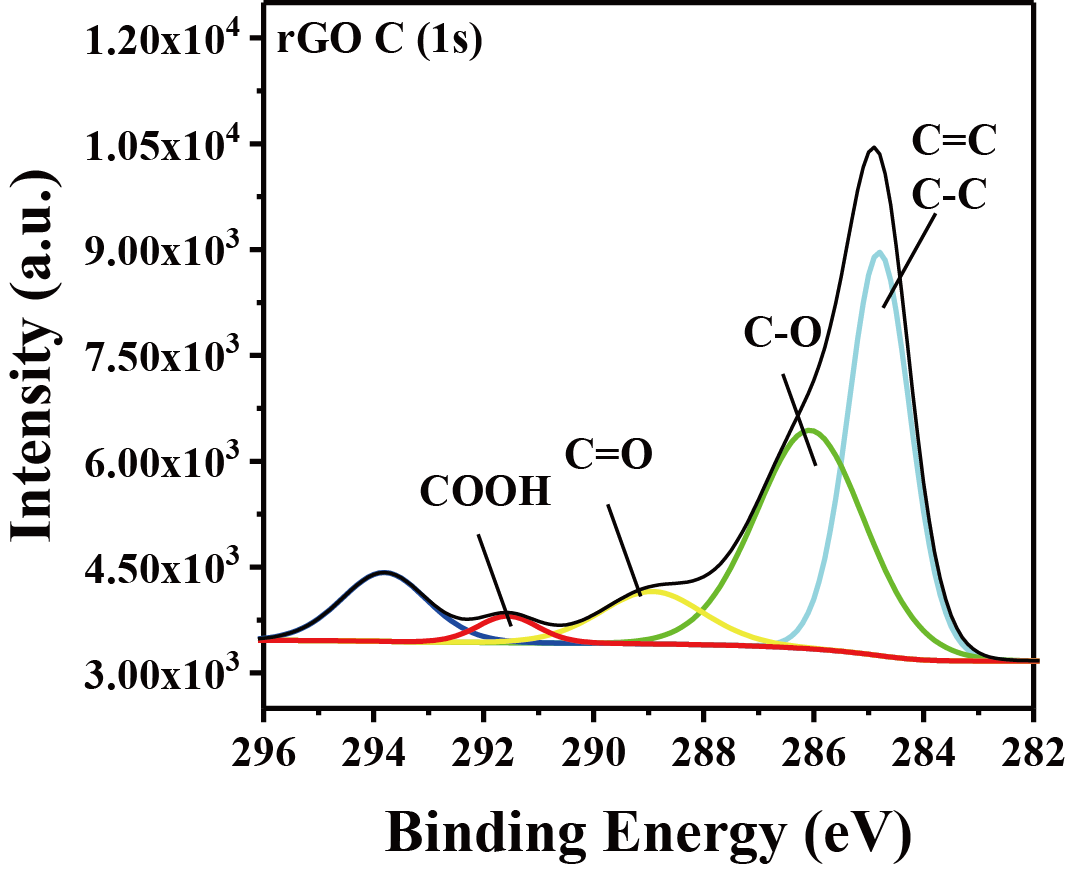


**Fig. S7** XPS spectra for the C 1*s* peaks of rGO nanosheets. The XPS spectra of the sample exhibit four peaks confirming the presence of functional groups.

Fig. S8 illustrates the XPS spectra for the C 1*s* peaks of CS and rGO/CS bilayer materials. No obvious changes are observed in C 1*s* peaks.


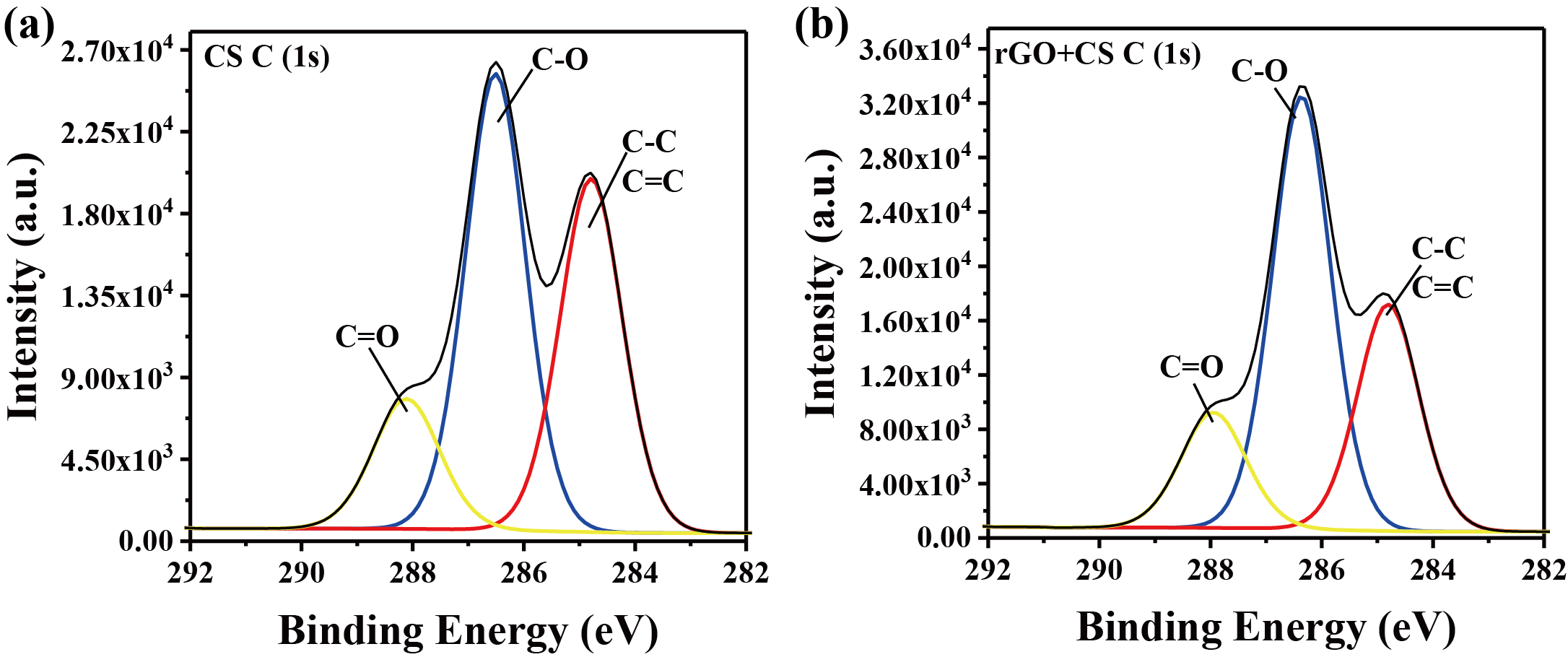


**Fig. S8** XPS spectra for the C 1*s* peaks of CS and rGO/CS samples.

Fig. S9 shows an increase in conductance of the memristor with the humidity of ambient changing from 30% to 70%, which is due to the increasing number of [ionized](javascript:;) protons with higher humidity. The behavior supports the hypothesis proposed in the manuscript that the current in the channel is formed due to the hopping of protons provided by CS through functional groups in rGO nanosheets.


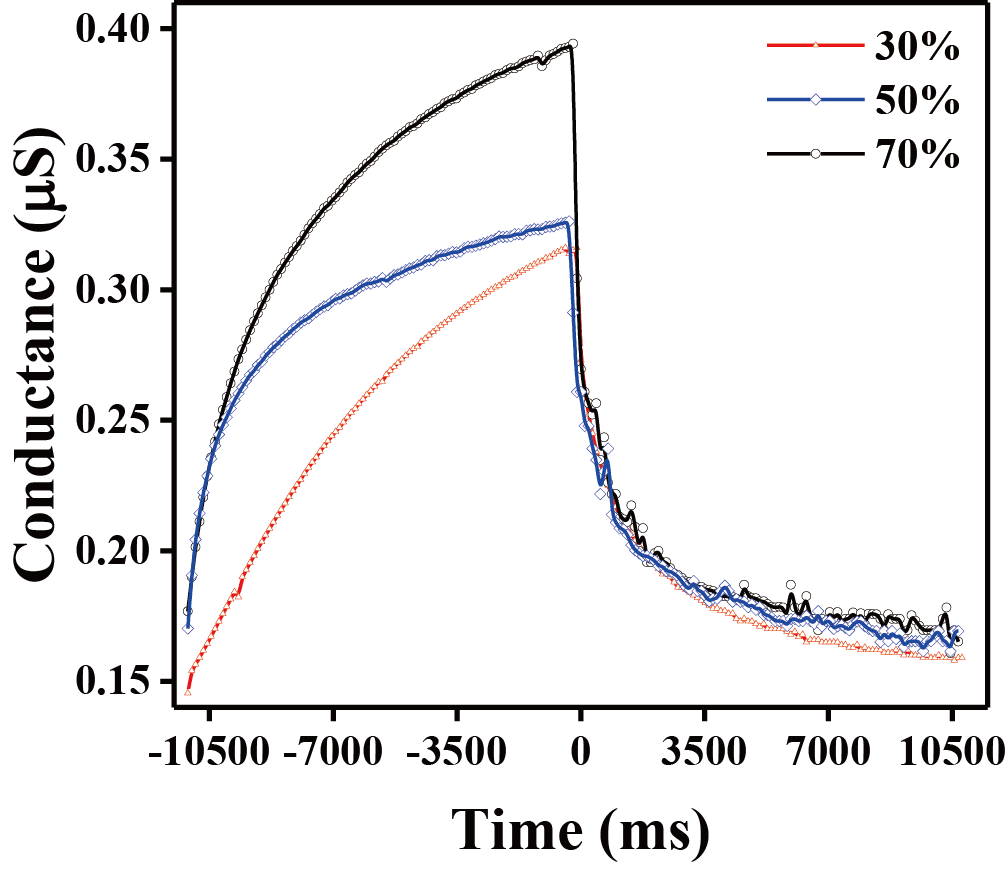


**Fig. S9** An increase in conductance of the memristor with the increasing humidity is observed.

Fig. S10a shows the UV-vis absorption spectra of rGO nanosheets. The corresponding square absorption energy (αh*v*, where α is the absorbance) against photo energy (h*v*) is demonstrated in Fig. S10b to determine the band gap of the material. From approximate linear extrapolation, a direct band gap of about 3.5 eV is obtained, which is consistent with previous reports .


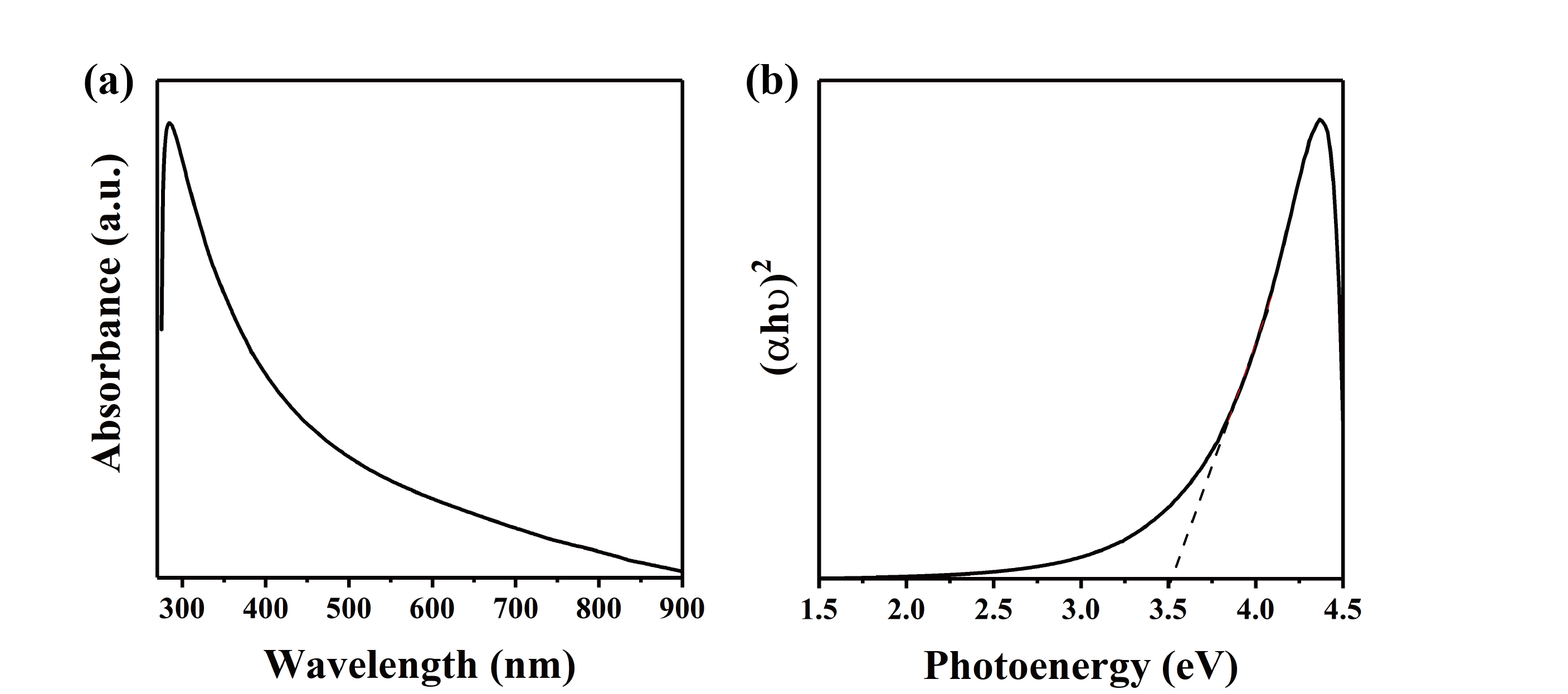


**Fig. S10 a** UV-vis absorption spectra of rGO nanosheets and **b** The corresponding square absorption energy against photo energy.

Fig. S11 shows the STP behavior of the artificial synapses repeating for five times, which confirms the stability of the device.


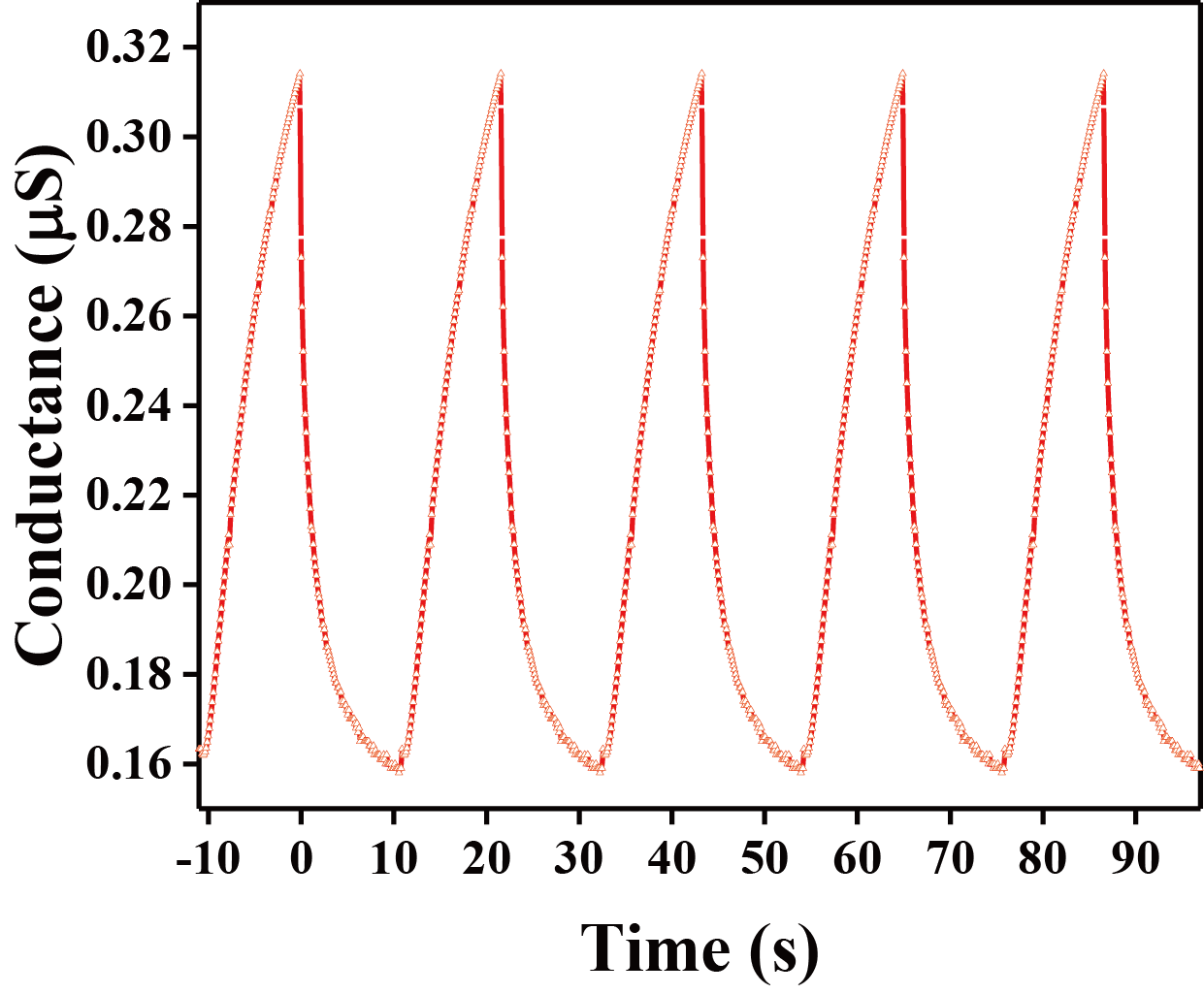


Fig. S11 Repetition of the STP behavior.
